# Mechanistic Sequence of Histone Deacetylase Inhibitors and Radiation Treatment: An Overview

**DOI:** 10.3390/ph17050602

**Published:** 2024-05-08

**Authors:** Elsie Neo Seane, Shankari Nair, Charlot Vandevoorde, Anna Joubert

**Affiliations:** 1 Department of Radiography, School of Health Care Sciences, Faculty of Health Sciences, University of Pretoria, Pretoria 0028, South Africa; 2 Department of Medical Imaging and Therapeutic Sciences, Faculty of Health and Wellness, Cape Peninsula University of Technology, Cape Town 7530, South Africa; 3 Radiation Biophysics Division, Separate Sector Cyclotron (SSC) Laboratory, iThemba LABS, Cape Town 7131, South Africa; shankari.nair.dr@gmail.com; 4 GSI Helmholtz Centre for Heavy Ion Research, Department of Biophysics, 64291 Darmstadt, Germany; c.vandevoorde@gsi.de; 5 Department of Physiology, School of Medicine, Faculty of Health Sciences, University of Pretoria, Pretoria 0028, South Africa; annie.joubert@up.ac.za

**Keywords:** histone deacetylase inhibitors, radiosensitisation, heterochromatin, double strand break, DNA repair

## Abstract

Histone deacetylases inhibitors (HDACis) have shown promising therapeutic outcomes in haematological malignancies such as leukaemia, multiple myeloma, and lymphoma, with disappointing results in solid tumours when used as monotherapy. As a result, combination therapies either with radiation or other deoxyribonucleic acid (DNA) damaging agents have been suggested as ideal strategy to improve their efficacy in solid tumours. Numerous in vitro and in vivo studies have demonstrated that HDACis can sensitise malignant cells to both electromagnetic and particle types of radiation by inhibiting DNA damage repair. Although the radiosensitising ability of HDACis has been reported as early as the 1990s, the mechanisms of radiosensitisation are yet to be fully understood. This review brings forth the various protocols used to sequence the administration of radiation and HDACi treatments in the different studies. The possible contribution of these various protocols to the ambiguity that surrounds the mechanisms of radiosensitisation is also highlighted.

## 1. Introduction

Histone deacetylase inhibitors (HDACis) have attracted a lot of interest as potential radiosensitisers that have selective effects on malignant cells with little effect on healthy cells [1,2]. The radiosensitising capabilities of HDACis in combination with photon irradiation have been well studied [3,4,5,6,7,8,9,10,11,12], while studies in combination with proton and carbon ion irradiation remain limited [13,14,15,16,17,18]. The exact mechanisms that underlie the radiosensitisation potential of these drugs for both photons and particle types of radiation also remain a matter of research. The effect of HDACis on the DNA damage repair (DDR) pathways as well as the effect they have on chromatin structure have been suggested as the main mechanisms [19,20,21,22,23,24]. However, the temporal sequence of HDACis in combination with radiation, as well as the optimal duration of HDACi treatment during a radiotherapy course, remains an elusive subject. As a result, different administration times and sequences have been used in in vitro studies so far, which makes the interpretation of results complex. Data on HDACi and radiation treatment from clinical studies and clinical trials are very limited [25,26,27,28]. In this review, the cellular effects of HDACis as well as the proposed mechanisms of radiosensitisation by HDACis are briefly reviewed, followed by the review of temporal sequences of radiation and HDACis used in different in vitro studies. The durations of incubation with HDACis before or after radiation in these studies are also reviewed.

## 2. Epigenetic Modulation by HDACs and HDAC Inhibitors

**Figure 1:**
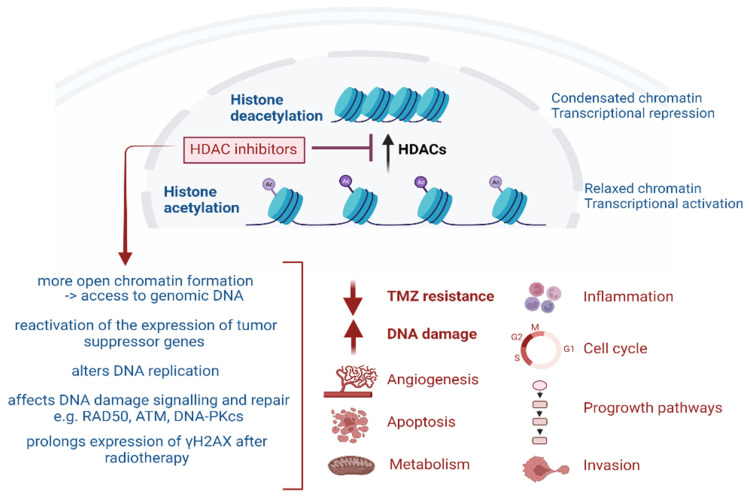
Overview of the broad effects of HDAC inhibitors [35].

During the process of malignant transformation in cells, genes that encode for histone acetyl transferases (HATs) can be amplified, translocated, or mutated leading to the inactivation of HATs. Consequently, histone deacetylase (HDACs) become overactive in malignant cells, resulting in the accumulation of deacetylated proteins. The overexpression of HDACs has been found in multiple human tumours such as lymphoma, prostate, gastric, leukaemia, colon, and breast [3,4,6,7,8,9,10,11,12,29]. HDACs have thus been recognised as promising targets to modify and reverse the aberrant epigenetic control in cancer cells [7]. HDACs are classified into four classes: Class I (HDACs 1, 2, 3, 8), Class II (HDACs 4, 5, 6, 7, 9, 10), Class III (Sirtuins1-7), and Class IV (HDAC 11) [30]. To this effect, HDACis have emerged as anti-cancer agents aimed at reversing the aberrant histone modification control in tumours [20,31,32]. The inhibition of HDAC activity results in the accumulation of acetylated proteins leading to cellular effects such as cell cycle arrest, differentiation, altered gene expression, and the inhibition of angiogenesis, metastasis, and apoptosis in a cell-type-dependent manner, as shown in Figure 1 [31,33,34].

The mechanism of action of HDACis has been linked to the structure and class of HDACis. In brief, HDACis are classified according to structure into benzamides (e.g., chidamide and entinostat), hydroxamic acids (e.g., vorinostat (SAHA), belinostat, panabinostat, and CUDC-101), cyclic tetrapeptides (e.g., romidepsin), and aliphatic acids (e.g., butyrate and valproic acid) [6,36,37]. Of these classes, hydroxamic acids are the main class that has been used and continues to be used in most studies [38]. Hydroxamic acids are preferred as they inhibit a broad range of HDACs (HDACs1-11), and they can cause cellular effects at low (nM) concentrations [37].

Earlier studies proposed histone hyper-acetylation and subsequent alterations in gene expression to be the main mechanism through which HDACis mediate their antiproliferative effect. Histone acetylation was reported to increase at 6 h post-treatment, reaching a maximum between 24 and 48 h after treatment with HDACi MS-275 in prostate carcinoma (DU145) and glioma (U251) cell lines [4]. However, this hypothesis could not explain the high specificity of HDACis for tumour cells. Subsequently, the hyper-acetylation of non-chromatin and non-histone proteins involved in cell death, proliferation, cell migration, inflammation, angiogenesis, cell cycle control, and DNA repair were acknowledged [13,32,33]. HDACi-induced cell death is mediated by several mechanisms, including apoptosis, autophagy, necrosis, and cell cycle arrest in a cell-type-dependent manner [31,33,34].

### 2.1. HDACi-Induced Apoptosis and Autophagy

The induction of apoptosis was initially recognised as the predominant mode of HDACi-induced cell death [39,40,41]. A number of studies reported HDACi-induced apoptosis through both the intrinsic and extrinsic pathways [20,31,32,33,39,42,43,44]. In particular, the intrinsic (mitochondria-related) apoptotic pathway has been supported by many studies as the main pathway that is activated by HDACi [20,31,33,39,42,43,44]. In brief, HDACi increases the production of reactive oxygen species (ROS), which leads to the loss of membrane potential. The loss of membrane potential enables cytochrome *c* to be released from the mitochondria to the cytoplasm, leading to the activation of caspase 9 and initiation of apoptosis [45]. The activation of both p53-dependent and -independent apoptotic pathways post-HDACi treatment has been reported, which would be beneficial for the treatment of p53 mutant tumours [2]. The role of the extrinsic apoptotic pathway and caspase-independent pathways in HDACi-induced apoptosis have long been acknowledged but remain poorly understood [32,39]. However, a possible link between autophagy and the extrinsic apoptotic pathway has been reported [46]. It is possible that since autophagic cell death in cancer is not well understood, some autophagic cell death may have previously been attributed to caspase-independent apoptotic death [47].

The role of autophagy in cancer is complex and remains controversial [48]. Traditionally, autophagy was regarded as a cell death mechanism which eliminates damaged organelles, proteins, macromolecules, and breakdown products from cells, thereby suppressing tumour progression. Hence, autophagy was referred to as cell death type II [2]. Later evidence suggested that autophagy can also act as a cell survival mechanism to promote tumour growth [49]. A conceivable explanation of the dynamic nature of autophagy in cancer elucidated that the role of autophagy depends on the stage, type of tumour, and genetic pre-disposition of the tumour [2,49,50]. In the early tumour stages, autophagy plays a protective role by preventing the accumulation of damaged organelles and macromolecules. In the late tumour stages, autophagy assumes the role of a survival mechanism by recycling degraded metabolites and counteracting the effect of chemotherapy treatment as well as oxygen and nutrient deprivation in hypoxic tumour areas, maintaining tumour growth [2,50]. It is appealing to associate the role of autophagy in late-stage tumours to the role of mammalian target of rapamycin (mTOR). mTOR plays a crucial role in metabolism and regulates autophagy by deactivating human autophagy initiation kinase ULK1, a component of upstream autophagic signalling pathway [2].

It comes as no surprise that HDACi-induced autophagy is also a highly debated topic. The proposed working mechanisms of HDACi-mediated autophagy include acetylation and upregulation of numerous autophagy-related proteins such as p53, p21, ATG3, ATG 7, ULK1, and Nuclear Factor kappa B (NF-ĸB). The inhibition of mTOR, transcription of FOX 01, inactivation of apoptosomes, upregulation of death-associated protein kinase (DAPK), and accumulation of reactive oxygen species (ROS) have also been suggested as possible mechanisms [2]. Several studies have reported a molecular shift between autophagy and apoptosis as well as dual induction of apoptosis and autophagy following HDACi treatment in different cell lines [2,30,47]. As an example, in chronic myeloid leukaemia (CLL), reduced activation of autophagy treatment with HDACi mocetinostat was reported, whereas the upregulation of autophagy was reported in MCF-7 cell line using the same HDACi [41,51]. Also, in HeLa cells, SAHA and sodium butyrate induced both autophagy and apoptosis [47]. The type of cell, genetic pre-disposition of the tumour, duration, and dose of HDCAis have been put forward as deciding factors of whether HDACi-induced autophagy acts as a pro-survival or pro-cell death mode [41]. If this stands true, this could in part explain the diverse results observed in different studies using different cell lines and different HDACi, as well as the uncertainty that surrounds the mechanisms of HDACi-induced cell death.

### 2.2. HDACi-Induced Upregulation of p21 and Cell Cycle Arrest

HDACi-induced apoptosis has been associated with the upregulation of cyclin-dependent kinase (CDK) inhibitor p21 and cell cycle arrest [2]. Transcriptional re-activation of p53 and the subsequent upregulation of p21 by HDACis have been reported in different cell lines [2,32,52]. Activation of p53 induces the expression of p21 to induce cell cycle arrest mainly in the gap 1 (G1) phase. An earlier report by Richon et al. alluded that HDACis are gene-specific after having observed only the upregulation of p21 and no alteration in the expression of either p27, also a CDK inhibitor, or γ-actin genes [53]. Cell cycle arrest in the G2/M phase of the cell cycle has also been reported and is accomplished by downregulating the expression of cyclin A by HDACis [31,54,55,56].

### 2.3. HDACi-Induced Inhibition of Angiogenesis

Studies on the effect of HDACis on angiogenesis remain limited. The inhibition of angiogenesis by HDACis was reported in nucleus polposus cells of interverbal discs, endothelial progenitor cells, human embryonic kidney (HEK) 293, and epithelial fibrosarcoma (HT1080) cells [57,58,59,60]. The inhibition of angiogenesis was evidenced by attenuation of vascular endothelial growth factor (VEGF), hyper-acetylation of hypoxia-inducible factor 1 (HIF-1α), and degradation of hypoxia-induced transcription factor [57,58,61]. Altered expression of pro- and anti-angiogenic genes following HDACi treatment has also been reported [60,62].

## 3. Radiosensitisation by HDAC Inhibitors

Evidence from pre-clinical studies has revealed that the combination of radiation and HDACis results in increased cell death in a number of cell lines, including lung, melanoma, prostate, glioma, colon, non-small cell lung cancer (NSCLC), osteosarcoma, and breast [3,4,5,6,7,8,9,10,11,12,14,15,17,18,63]. When used as monotherapy, HDACis have shown promising therapeutic outcomes in haematological malignancies such as leukaemia, multiple myeloma, and lymphoma, with disappointing results in solid tumours [20,31,37,64,65]. The molecular basis for the poor clinical outcomes in solid tumours is still unclear but is thought to be due to the short drug half-life of HDACis, which leads to poor drug distribution, poor HDAC isoform selectivity, and poor patient selection [13,38]. HDACi-induced radiosensitisation is mainly attributed to their role in DNA damage response (DDR) and their effect on chromatin structure [12]. As a result, combination therapies either with radiation or other DNA-damaging agents have been suggested as an ideal strategy to improve their efficacy in solid tumours [5,14,36].

### 3.1. DNA DSB Induction and DNA Damage Repair (DDR)

Following the induction of DNA, double-strand breaks by radiation pathways that sense, respond, and repair the damage are activated [66]. DNA double-strand breaks are repaired using two basic mechanisms, homologous recombination (HR) or non-homologous end joining (NHEJ). During the initial stages of both HR and NHEJ, ATM promotes the processing of the broken DNA ends by the MRE11/NBS/Rad50 (MRN) complex to resect the broken ends into 3′ DNA single-strand tails [67]. The choice of repair pathway is dictated in part by the presence or absence of p53 binding protein 1 (53BP1). In the presence of 53BP1, HR is inhibited and NHEJ is initiated. During NHEJ, Ku70 and Ku80 heterodimer bind to the DNA ends and block the resection of the 5′ end. Ku70/80 recruits DNA PKs to the broken ends. In the final steps, the DNA PK complex recruits a ligase complex consisting of X-ray repair complementing defective in Chinese Hamster 4 (XRCC4), XRCC4-like factor-DNA ligase 4 (XLF-LIGIV), and polynucleotide kinase (PNK) to perform the ligation of broken ends [21,68]. NHEJ is an error-prone mechanism which is active throughout the cell cycle, mainly in the G1 phase [21,69].

During HR, the damaged DNA ends are resected by the Mre11-Rad50-Nbs1(MRN) complex to form 3′prime ends. The 3′prime ends are coated by replication protein A (RPA) to form a nucleoprotein filament to which HR proteins (breast cancer tumour suppressor (BRCA1), RAD51, and RAD52) can bind [66]. RAD51 is a key protein in HR that facilitates strand exchange with the complementary undamaged DNA strand to form the holiday junction. The resolution of the holiday junction into two DNA duplexes is carried out by MMS4 and MUS81 [68]. HR requires the presence of an undamaged DNA strand (sister chromatid or chromosomes) to use as a template for repair. Sister chromatids are only available during the late S-and G2 phases after DNA replication, thus HR is active during these phases. The use of a DNA template for repair facilitates accurate repair which makes HR an error-free pathway [68,69]. HDACis have been observed to repress DNA repair proteins such as MRE11/Rad50/NBS1 (MRN) complex and Rad51 involved in HR and ku70, ku80, DNA PK involved in NHEJ [12,19,22,70].

### 3.2. Role of HDACs and HDACis in the Early Stages of the DNA Damage Response (DDR)

**Figure 2:**
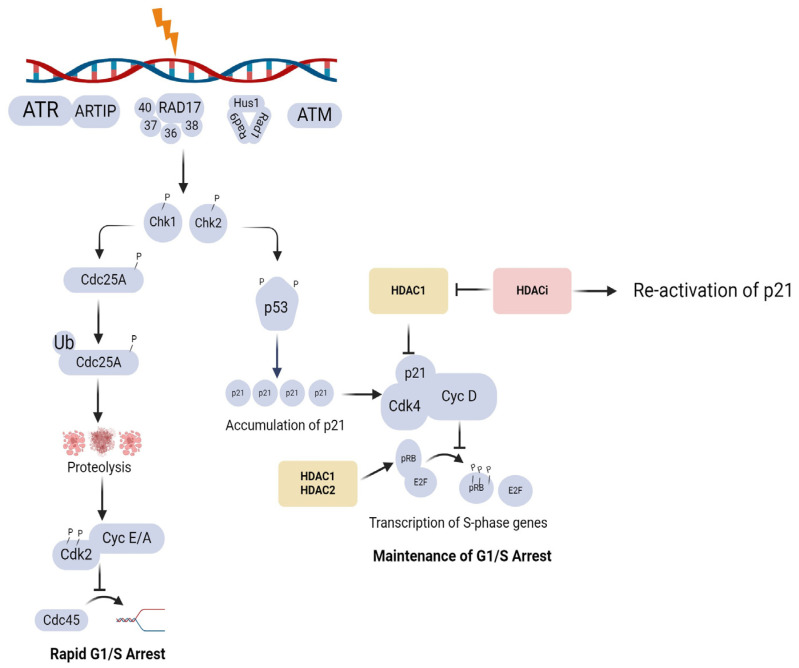
Illustration of the role of HDACs in cell cycle regulation (created with BioRender.com https://app.biorender.com/illustrations/6420e9f530ff29832d070b3f (accessed 23 April 2024).

The DDR consists of a complex network of signalling pathways that involves the activation of cell cycle checkpoints, DNA repair, transcriptional programmes, and programmed cell death [71]. Cell cycle checkpoints monitor the progression of the cell cycle by stopping entry into S-phase (G1/S checkpoint), delaying S-phase progression (intra-S checkpoint), or preventing entry into mitosis (G2/M checkpoint) in response to DNA damage by exogenous agents such as radiation or due to replication stress [71]. At the G1/S checkpoint, ataxia-telengiectasia mutated (ATM) is autophosphorylated and initiates the DNA damage signalling cascade by activating Chk 2. Chk2 phosphorylates cell division cycle (CDC)25A phosphatase, which inhibits the activation of Cyclin E/A and its binding to Cdk 2 to induce rapid cell cycle arrest. At the G2/M checkpoint, ATR is autophosphorylated and initiates Chk1 which phosphorylates (CDC)25A, -B, and -C phosphatases. The maintenance of cell cycle arrest is promoted by the phosphorylation of p53 by Chk 2, which in turn induces the accumulation of Cdk inhibitor p21. The binding of p21 to the cyclin D and Cdk 4 complex keeps the retinoblastoma protein (pRb) in an unphosphorylated state and promotes its association with the E2F1 transcription factor, maintaining cell cycle arrest, as shown in Figure 2. HDAC1 has been reported to repress p21, and the inhibition of HDAC1 activity, therefore, activates p21 to induce and maintain cell cycle arrest [72]. Earlier studies have also pointed out that the interaction between HDAC1/2 and E2F transcription factors is important for the G1-S cell cycle transition [73,74]. A recent study conducted in colon cancer (HCT116) cells reported that the pRb-E2F complex does not necessarily require HDAC activity to induce rapid cell cycle arrest, but HDAC activity might be required for complete cell cycle arrest and to maintain arrest [75].

Evidence suggests that HDACs play a role in regulating ATM. HDAC1 was observed to interact with ATM, particularly after exposure to gamma-radiation in fibroblast cells [76]. In support of this observation, the reduced activation of ATM after treatment with HDACi SAHA was reported in breast (MCF-7, T-47D), melanoma (SK-MEL-28), human osteosarcoma (Saos-2), and A549 cell lines [76]. This observation was further corroborated by reports of accumulation of HDAC1 and 2 at the damaged sites within 5 min of DNA damage induction and dissociation at 30 min after radiation treatment [77]. The rapid accumulation and dispersal of HDAC1 and 2 were associated with the rapid deacetylation of Histone3 lysine 56 (H3K56) and Histone4 lysine 16 (H4K16) which favours non-homologous end-joining (NHEJ), followed by histone acetylations that favours homologous repair (HR) [77]. The authors also observed that the acetylation levels of H3K56 were reduced upon the induction of DNA damage without treatment with HDACis [77]. HDAC1 and 2 bind to CDK inhibitors p21 and p27, thereby reducing their activity and resulting in cell cycle progression from G1 to S-phase [39,40,42]. The inhibition of HDAC1, therefore, restores the activity of p21, leading to G1 cell cycle arrest [40]. The depletion of HDAC1 was also reported to partially contribute to G2/M arrest and cell cycle arrest [40,43]. HDAC4 was reported to co-localise with another DDR indicator, 53BP1, at DSB sites in fibroblast (FT169A and YZ5 cells). Furthermore, DNA damage-induced G2 checkpoint was inactivated and the levels of 53BP1 were observed to be reduced when HDAC4 was knocked down, leading to the conclusion that HDAC4 is critical in maintaining G2 checkpoint [47,78]. The roles of the different HDACs in the DDR are summarised in Table 1.

## 4. Impact of Chromatin Structure on Radiation Response

The architecture of chromatin during DNA damage induction, recognition, signalling, and repair is an important factor that can dictate the successful repair or misrepair of DNA double-strand breaks [23]. However, the topic has received little attention over the years, partly due to a lack of efficient in vitro systems for the manipulation of long chromatin and quantitative detection for DSBs [66]. As early as 1991, Smerdon proposed the “access-repair-restore” model to describe the impact of chromatin structure on DNA repair. In brief, the model proposed that in order for DNA damage to be repaired, DNA damage in different chromatin structures should be detected, and local chromatin structure needs to be remodelled to allow repair proteins to have access to damaged sites and to be restored after repair [86].

For the purposes of this review, a brief description of chromatin structure is justified. Chromatin is organised in structures named nucleosomes. Each nucleosome consists of DNA wrapped around histone octamer which consists of histones, H2A, H2B, H3, and H4. The nucleosome is then connected to a linker DNA and histone H1 [87]. The amino terminus tails of the histones protrude from the histone core and are open to various histone post-translational modifications such as acetylation, ubiquitination, or methylation. The two forms of chromatin, heterochromatin and euchromatin, are regulated by post-translation modification (PMT) of histones. Some of the post-translational modifications that occur on the histone tails play an important role in the DDR [87,88].

### 4.1. Influence of Chromatin Structure on DNA Damage Induction, Detection, and Repair

It has long been accepted that heterochromatin has a protective effect on DNA against ionising radiation [23,24,89]. Cowel et al. observed that γ-H2AX foci, a marker of DSB, were absent from areas which contained heterochromatin markers HP1α and H3K9Me3 in the nuclei of MCF-7 cells [90]. Similarly, Kim et al. reported an increased number of γ-H2AX foci in areas of open chromatin [91]. A similar observation was made by Takata et al., who reported a 5–50-fold decrease in DSB induction by γ-rays in condensed chromatin as compared to decondensed chromatin [23]. Clearly, there is agreement that heterochromatin confers protection against DNA DSB induction by radiation. However, the underlying mechanisms around this protective effect remain a matter of debate. Warters et al. argued that the protective effect against radiation is dependent on the level of chromatin-associated non-histone proteins in heterochromatin rather than on the concentration of chromatin [89]. In other words, the more proteins carried by the chromatin, the higher the protection of chromatin from ionising radiation. The authors observed a 70 times higher yield of DSB in deproteinised DNA as compared to intact nuclei. Elia and Bradley concluded that chromatin domains that differ in tertiary structure and protein composition may also differ in their susceptibility to DNA double-strand breaks induced by ionising radiation [23]. However, in both studies, the protective effect offered by chromatin compaction was acknowledged.

Nygren et al. investigated the role of DNA-bound proteins in the protective effect and they reported an increase in the protective effect of a factor of 14 in single-strand breaks and a factor of 5 in double-strand breaks when DNA-bound proteins were removed [92]. They concluded that DNA-bound proteins protect chromatin to a limited extent by acting as free radical scavengers. The greater part of the protection was attributed to the fact that DNA in the chromatin is made up of large, compact aggregates where the distance between separate aggregates exceeds the effective range of the hydroxyl (·OH) radicals. Further, inside the large aggregates, the amount of DNA damaging ·OH radicals produced is less due to reduced water content as compared to when DNA is more evenly distributed in a given volume [92]. Similarly, Takata et al. observed that the level and composition of proteins were similar between condensed and decondensed chromatin in Hela cells, and concluded that the protective effect is due to the concentration of chromatin and not the level of chromatin-associated proteins as previously proposed [23]. They reasoned that a lower chromatin concentration contains more water molecules, with subsequent increases in reactive radicals that are formed. It remains contentious whether the opposing observations between Takata et al. and Warters et al. could be due to the different chromatin manipulation methods used in the respective studies [23,89].

### 4.2. DNA Damage Induced Chromatin Modifications

Evidence from earlier biochemical studies pointed out that the induction of double-strand breaks causes the remodelling of chromatin structure around the damaged site [87,93]. Subsequently, Lisby et al. reported that in yeast *Saccharomyces cerevisiae*, DNA DSB localises to repair foci which contain Rad52 protein, suggesting that multiple DSBs can be repaired by a Rad52 repair foci [94]. These findings implied that the mobility of chromatin allows DSBs to localise at one repair site [94,95]. The authors, however, acknowledged that the localisation and interaction of DSB observed in the yeast *Saccharomyces cerevisiae* might be due to the small nuclear size in yeast as compared to the nuclei of mammalian cells, rather than due to the mobility of chromatin. Also, as compared to mammalian cells, homologous recombination is a dominant repair mechanism in yeast, which would explain the co-localisation of Rad52 foci in yeast and not in mammalian cells [93,94]. Similarly, in a later study, the relocalisation of DSB to the nuclear periphery before the recruitment of Rad51 was reported in Drospohlilla [96].

A contradictory observation was made by Kruhlak et al. in mammalian cells. The authors noted that remodelling of chromatin architecture at DSB sites does not involve large-scale mobility of chromatin to assemble at repair centres but rather small-scale mobility in the micrometre range [93]. The authors reported chromatin expansion at 20 s after irradiation which lasted up to 180 s after UV irradiation in HeLa cells. In an attempt to explain the local expansion in chromatin that was observed in the area around the DSB, the authors conceptualised that after DSB formation, the break causes the damaged chromatin region to unfold, relieving the torsional stress exerted by the packaging of DNA, thus resulting in the expansion and relaxation of chromatin. They concluded that the observed chromatin relaxation and expansion might be due to the presence of DSB sensor proteins which exhibit chromatin unwinding properties [93]. Indeed, the increased acetylation of DDR proteins histones H2A and H4 at DSB sites was reported to lead to the rapid formation of open chromatin by a number of studies [97,98]. Perhaps, the reasoning offered by Takata et al., that chromatin relaxation after DSB induction was part of the evolutionary conservation of the genome to allow access for repair protein, should be given consideration [23].

Another important observation made was an increase in the size of foci as chromatin becomes open [91]. A reasonable explanation was later offered by Kruhlak et al. that the time-dependent increase in the size of individual foci which was noted was not due to the merging of multiple DSBs as a result of the mobility of chromatin, but rather due to the spreading of H2AX phosphorylation over large chromatin area, which subsequently acts as a docking site for DNA damage proteins [93]. An akin reasoning was given by Krawczyk et al. that the mobility of foci might simply be due to the relaxation of chromatin and not due to the mobility of chromatin [99].

### 4.3. Chromatin Modification and Type of Radiation

The protective effect of heterochromatin against DSB induction has been linked to the type of radiation, i.e., low or high linear energy transfer (LET) radiation [23]. Earlier reports associated the protective effect with low LET radiation. This was because the radiolysis of water molecules with subsequent formation of hydroxyl radicals has long been acknowledged as the major contributor to DNA damage, particularly when low LET radiation is used. It was therefore argued that since heterochromatin has fewer water molecules per chromatin, fewer hydroxyl radicals are produced, as discussed in Section 4.2. The opposite is true for decondensed chromatin [23]. However, Takata et al. reported that heterochromatin protects against DNA damage not only from low LET radiation but from heavy ions as well. The authors made this conclusion after observing a 7-fold increase in radioprotection in heterochromatin when carbon ion was used in HeLa cells [23]. Furthermore, using Monte Carlo simulations, the complexity of DNA damage induction caused by low and high LET types of radiation was observed to be the same in heterochromatin, as well as in euchromatin. However, inefficient repair was noted in heterochromatin [23,87]. This lends support to the arguments presented by Takata et al. that the protective effect of heterochromatin hinders the efficient repair of DSB [23].

## 5. Sequencing of HDACi Treatment and Radiation

The use of HDAC inhibitors in combination with radiation therapy remains to be a matter of ongoing research. Evidence from numerous studies suggests that there is agreement on HDACi treatment before radiation (pre-irradiation HDACi protocol), with only a few studies having investigated the HDACi post-irradiation (post-irradiation HDACi protocol) [7,100,101]. In the studies using pre-irradiation HDACi protocols, HDACi treatment was given at different timepoints (3, 6, 16, 18, 24, and 48 h) before irradiation in different studies [7,10,11,14,17,18,63,70,102,103,104,105,106,107,108]. Kim et al. determined that 18 and 24 h of pre-irradiation incubation of the A549 cell line with trichostatin A (TSA) resulted in enhanced radiation sensitisation to 2–8 Gy X-rays as compared to HDACi treatment at 3, 6, and 12 h post-irradiation [100]. In U251 glioma cell lines, dose enhancement factors of 1.38, 1.4, and 1.46 were reported when cells were exposed to 1.5 mmol/L valproic acid (VPA) at 6 h, 24 h, and immediately after irradiation, respectively [6]. A greater dose enhancement factor of 1.71 was noted with a 16 h pre- and post-incubation in VPA leading to a conclusion that pre-and post-exposure to HDACis is necessary for maximal radiosensitisation [7]. It is noteworthy to mention that a higher dose enhancement factor (1.46) was reported when VPA was administered immediately after radiation, as compared to when VPA was administered 6 h and 24 h post-irradiation. It is tempting to speculate that the modestly higher dose enhancement factor might be in part due to radiation-induced chromatin modifications. As set forth by numerous studies [24,86,87,88,91,96], following the induction of DSB by radiation, chromatin relaxation around the DSB occurs. Therefore, one could mechanistically reason that the addition of HDACis immediately post-IR, coincides with the rapid chromatin changes that occur post-irradiation. In support, Kruhlak et al. reported chromatin changes as early as 20 s after irradiation [93].

Van Nifterik reported a dose enhancement factor of 1.3 and 1.4 for D384 medulloblastoma cells and 1.7 and 1.5 for T98 glioblastoma cells for 24 and 48 h pre-incubation periods with 5 mM VPA [101]. The authors further reported that they did not observe any enhancement, with dose enhancement factors of 1.1 for D384 cells and 1.0 for T98 cells, when cells were incubated in VPA for 24 h post-irradiation only. It is tempting to speculate that the difference in radiosensitivity between the pre-and post-irradiation HDACi protocols may in part be due to two important factors, i.e., different plating methods, (pre-irradiation plating (pre-IR plating) and post-irradiation plating (post-IR plating)) and the incubation period in HDACis. Typically, radiation sensitivity studies are conducted using colony survival assays [109]. In the pre-IR plating setting, cells are seeded, allowed to attach, and treated. In the post-IR setting, cells are treated followed by trypsinisation, and the required numbers of cells are seeded in plates. In addition, the post-IR plating method has two methods that can be used, immediate plating (IP) and delayed plating (DP). In the IP method, cells are seeded immediately after radiation, and in the DP method, cells are seeded hours after irradiation. Of the two post-IR plating methods, IP was observed to exhibit a lower survival than DP [110,111]. The difference between the resultant cell survival curves when using the two methods was explained by the cell’s capacity to repair potentially lethal damage [110,111]. Moreover, in most studies, it is not specified whether DP or IP was used, which also poses a challenge for data integration. Oike et al. reported consistent SF_2_, SF_4_, SF_6_, SF_8_, SF_10_, D_10_, and D_50_ values between pre-IR plating and post-IR plating methods, using lung cancer (A549) and submandibular gland (HSG) cells [109]. The study, however, did not investigate the possible impact of delayed platting in the post-IR plating setting [109]. It remains undetermined whether consistent results observed by Oike et al. can extrapolate to other cell lines, and most importantly, in the context of this review, whether the results can be applied to the combination treatment of HDACis and radiation.

Furthermore, whether a post-IR HDACi protocol or pre-IR HDACi protocol is used, the total period of HDACi incubation used in the various studies is different. As an example, Chinnaiyan et al. evaluated both pre- and post-IR HDACi protocols. In the post-IR HDACi protocol, cells were seeded in 6-well plates, and VPA was added immediately 6 h and 24 h after irradiation. Cells were incubated in VPA-containing media for the remainder of the assay [7]. When using the pre-irradiation HDACi protocol, the cells were treated with VPA for 16 h, irradiated, and rinsed with PBS before fresh HDACi-free media was added. In the pre- and post-protocol, cells were pre-treated with VPA for 16 h and returned for incubation post-IR. The authors reported improved radiosensitisation (factor of 1.7) when cells were exposed to VPA pre- and post-irradiation, as compared to factors of 1.3 when VPA was administered pre-IR only. This suggests that the removal of VPA-containing media at plating could be the reason for the observed non-enhancement. Also, taking into account the modes of HDACi-induced cell death in Section 2.1, HDACi-induced autophagy is reported to depend on the duration and dose of HDACis [2]. It is enticing to speculate that different periods of HDACi incubation, as well as different HDACi concentrations noted in the different studies, might have played a role in the mode of cell death, with subsequent differences in the observed results.

## 6. Conclusions

The combination therapy of HDACis and radiation is complex. The matter is further complicated by the pleiotropic effects of HDACis on histone and non-histone targets in cells. The biological rationale for this combination therapy relies on the ability of HDACis to modulate epigenetics to maximise the radiation effect. Mechanistically, pre-IR HDACi treatment induces chromatin relaxation to facilitate increased DSB induction by radiation. However, from existing reports, different temporal sequencing protocols of HDACis and radiation have been used. Some studies employed the pre-IR HDACi protocol and others used post-IR HDACi protocol. The different plating methods, i.e., pre-IR or post-IR plating, delayed plating, or immediate plating, also pose a challenge with the integration of data from different studies. Considering the ability of radiation and HDACis to modify chromatin structure, as well as the paradoxical relationship between apoptosis and autophagy under different conditions, the molecular interplay of the two modalities is bound to be complex.

Several reports have emphasised that HDACi treatment depends on the cell type, period of incubation with HDACis, as well as the dose of HDACi. To date, evidence suggests that incubation for 24–48 h pre-irradiation is the most optimal sequence. The mechanisms involved remain elusive. The inhibition of DNA DSB repair has traditionally been hailed as the main mechanism of HDACi-induced radiosensitisation; however, emerging evidence from different cell lines suggests otherwise. For example, numerous studies reported that HDACis impair DSB repair as evidenced by the prolonged appearance of the γ-H2AX foci. However, Moertl et al. reported not having observed any prolongation of foci after treatment with SAHA and CUDC-101 in pancreatic cell lines (Su.86.86, MIA Paca-2, and T3M-4) [100]. Clearly, more studies using different cell lines and different HDACis are required to fully unravel the mechanisms of radiosensitisation. For future studies, analysis of DSB repair proteins in addition to the appearance of γ-H2AX foci, as well as investigation of other modes of cell death such as autophagy and ROS production, may aid in fully elucidating the mechanisms involved. In view of the complex mechanism of action of HDACis under different conditions, it seems reasonable to recommend that the optimal temporal sequencing protocol of HDACi and radiation, as well as the optimal period of HDACi incubation, should be first determined for each cell and each HDACi used for in vitro studies. Mechanistically, this would allow sufficient time for chromatin to be remodelled to allow increased DSB induction by radiation. For combination therapy of HDACi and radiation in the clinic, it is also recommended that HDACi be administered hours before radiation.

## Figures and Tables

**Table 1:** Roles of HDACs in the DDR.

HDAC	Role	Reference
HDAC 1 and 2	DNA-damage signalling	
	Stabilise broken ends during NHEJ	[77]
	Influence persistence of Ku70 and Artemis at DNA damage sites- promoting NHEJ	[13]
	Deactivates the function of p21 and p27	[79,80]
	Hypo-phosphorylation of RB gene	[81]
HDAC 3	Maintenance of chromatin structure and genomic stability	[82]
	Essential for DNA DSB repair	[13]
	Recruits Xeroderma Pigmentosum C (XPC) during Nucleotide excision repair (NER)	[83]
HDAC 4	Silencing of chromatin near broken ends.	[78]
	Co-localises with 53BP foci, and contributes to the stability of 53BP	[13]
HDAC 6	Reduces cellular sensitivity to damaging agents	[84]
	Repair of DNA mismatch	[13]
HDAC 9 and 10	DSB repair via the HR pathway	[13]
	G2/M transition regulates transcription of cyclin A2	[80]
SIRT1	Reduces activity of p53 Modulation of γ-H2AX foci, BBRCA1, Rad51, and NBS foci formation	[85]
SIRT6	Facilitates DSB repair by activating PARP1	[80]

## Data Availability

Data sharing is not applicable.

## References

[1] Lee J.H., Choy M.L., Ngo L., Foster S.S., Marks P.A. (2010). Histone deacetylase inhibitor induces DNA damage, which normal but not transformed cells can repair. Proc. Natl. Acad. Sci. USA.

[2] Mrakovcic M., Bohner L., Hanisch M., Fröhlich L.F. (2018). Epigenetic Targeting of Autophagy via HDAC Inhibition in Tumor Cells: Role of p53. Int. J. Mol. Sci..

[3] Camphausen K., Scott T., Sproull M., Tofilon P.J. (2004). Enhancement of xenograft tumor radiosensitivity by the histone deacetylase inhibitor MS-275 and correlation with histone hyperacetylation. Clin. Cancer Res..

[4] Camphausen K., Tofilon P.J. (2007). Inhibition of histone deacetylation: A strategy for tumor radiosensitization. J. Clin. Oncol..

[5] Groselj B., Sharma N.L., Hamdy F.C., Kerr M., Kiltie A.E. (2013). Histone deacetylase inhibitors as radiosensitisers: Effects on DNA damage signalling and repair. Br. J. Cancer.

[6] Damaskos C., Garmpis N., Valsami S., Kontos M., Spartalis E., Kalampokas T., Kalampokas E., Athanasiou A., Moris D., Daskalopoulou A. (2017). Histone Deacetylase Inhibitors: An Attractive Therapeutic Strategy Against Breast Cancer. Anticancer Res..

[7] Chinnaiyan P., Cerna D., Burgan W.E., Beam K., Williams E.S., Camphausen K., Tofilon P.J. (2008). Postradiation sensitization of the histone deacetylase inhibitor valproic acid. Clin. Cancer Res..

[8] Schlaff C.D., Arscott W.T., Gordon I., Tandle A., Tofilon P., Camphausen K. (2013). Radiosensitization Effects of Novel Triple-Inhibitor CUDC-101 in Glioblastoma Multiforme and Breast Cancer Cells In Vitro. Int. J. Radiat. Oncol. Biol. Phys..

[9] Chiu H.W., Yeh Y.L., Wang Y.C., Huang W.J., Chen Y.A., Chiou Y.S., Ho S.Y., Lin P., Wang Y.J. (2013). Suberoylanilide hydroxamic acid, an inhibitor of histone deacetylase, enhances radiosensitivity and suppresses lung metastasis in breast cancer in vitro and in vivo. PLoS ONE.

[10] Baschnagel A., Russo A., Burgan W.E., Carter D., Beam K., Palmieri D., Steeg P.S., Tofilon P., Camphausen K. (2009). Vorinostat enhances the radiosensitivity of a breast cancer brain metastatic cell line grown in vitro and as intracranial xenografts. Mol. Cancer Ther..

[11] Chen X., Wong P., Radany E., Wong J.Y. (2009). HDAC inhibitor, valproic acid, induces p53-dependent radiosensitization of colon cancer cells. Cancer Biother. Radiopharm..

[12] Munshi A., Kurland J.F., Nishikawa T., Tanaka T., Hobbs M.L., Tucker S.L., Ismail S., Stevens C., Meyn R.E. (2005). Histone deacetylase inhibitors radiosensitize human melanoma cells by suppressing DNA repair activity. Clin. Cancer Res..

[13] Antrobus J., Parsons J.L. (2022). Histone Deacetylases and Their Potential as Targets to Enhance Tumour Radiosensitisation. Radiation.

[14] Gerelchuluun A., Maeda J., Manabe E., Brents C.A., Sakae T., Fujimori A., Chen D.J., Tsuboi K., Kato T.A. (2018). Histone Deacetylase Inhibitor Induced Radiation Sensitization Effects on Human Cancer Cells after Photon and Hadron Radiation Exposure. Int. J. Mol. Sci..

[15] Barazzuol L., Jeynes J.C., Merchant M.J., Wéra A.C., Barry M.A., Kirkby K.J., Suzuki M. (2015). Radiosensitization of glioblastoma cells using a histone deacetylase inhibitor (SAHA) comparing carbon ions with X-rays. Int. J. Radiat. Biol..

[16] Johnson A.M., Bennett P.V., Sanidad K.Z., Hoang A., Jardine J.H., Keszenman D.J., Wilson P.F. (2021). Evaluation of Histone Deacetylase Inhibitors as Radiosensitizers for Proton and Light Ion Radiotherapy. Front. Oncol..

[17] Yu J.I., Choi C., Shin S.W., Son A., Lee G.H., Kim S.Y., Park H.C. (2017). Valproic Acid Sensitizes Hepatocellular Carcinoma Cells to Proton Therapy by Suppressing NRF2 Activation. Sci. Rep..

[18] Choi C., Lee G.H., Son A., Yoo G.S., Yu J.I., Park H.C. (2021). Downregulation of Mcl-1 by Panobinostat Potentiates Proton Beam Therapy in Hepatocellular Carcinoma Cells. Cells.

[19] Groselj B., Kerr M., Kiltie A.E. (2013). Radiosensitisation of bladder cancer cells by panobinostat is modulated by Ku80 expression. Radiother. Oncol..

[20] Jenke R., Ressing N., Hansen F.K., Aigner A., Buch T. (2021). Anticancer Therapy with HDAC Inhibitors: Mechanism-Based Combination Strategies and Future Perspectives. Cancers.

[21] Maier P., Hartmann L., Wenz F., Herskind C. (2016). Cellular Pathways in Response to Ionizing Radiation and Their Targetability for Tumor Radiosensitization. Int. J. Mol. Sci..

[22] Shabason J.E., Tofilon P.J., Camphausen K. (2011). Grand rounds at the National Institutes of Health: HDAC inhibitors as radiation modifiers, from bench to clinic. J. Cell. Mol. Med..

[23] Takata H., Hanafusa T., Mori T., Shimura M., Iida Y., Ishikawa K., Yoshikawa K., Yoshikawa Y., Maeshima K. (2013). Chromatin Compaction Protects Genomic DNA from Radiation Damage. PLoS ONE.

[24] Venkatesh P., Panyutin I.V., Remeeva E., Neumann R.D., Panyutin I.G. (2016). Effect of Chromatin Structure on the Extent and Distribution of DNA Double Strand Breaks Produced by Ionizing Radiation; Comparative Study of hESC and Differentiated Cells Lines. Int. J. Mol. Sci..

[25] Chan E., Arlinghaus L.R., Cardin D.B., Goff L., Berlin J.D., Parikh A., Abramson R.G., Yankeelov T.E., Hiebert S., Merchant N. (2016). Phase I trial of vorinostat added to chemoradiation with capecitabine in pancreatic cancer. Radiother. Oncol..

[26] Galanis E., Anderson S.K., Miller C.R., Sarkaria J.N., Jaeckle K., Buckner J.C., Ligon K.L., Ballman K.V., Moore D.F., Nebozhyn M. (2017). Phase I/II trial of vorinostat combined with temozolomide and radiation therapy for newly diagnosed glioblastoma: Results of Alliance N0874/ABTC 02. Neuro-Oncol..

[27] Gurbani S.S., Yoon Y., Weinberg B.D., Salgado E., Press R.H., Cordova J.S., Ramesh K.K., Liang Z., Vega J.V., Voloschin A. (2019). Assessing Treatment Response of Glioblastoma to an HDAC Inhibitor Using Whole-Brain Spectroscopic MRI. Tomography.

[28] Teknos T.N., Grecula J., Agrawal A., Old M.O., Ozer E., Carrau R., Kang S., Rocco J., Blakaj D., Diavolitsis V. (2019). A phase 1 trial of Vorinostat in combination with concurrent chemoradiation therapy in the treatment of advanced staged head and neck squamous cell carcinoma. Investig. New Drugs.

[29] Zhang C., Richon V., Ni X., Talpur R., Duvic M. (2005). Selective induction of apoptosis by histone deacetylase inhibitor SAHA in cutaneous T-cell lymphoma cells: Relevance to mechanism of therapeutic action. J. Investig. Dermatol..

[30] Passaro E., Papulino C., Chianese U., Toraldo A., Congi R., Del Gaudio N., Nicoletti M.M., Benedetti R., Altucci L. (2021). HDAC6 Inhibition Extinguishes Autophagy in Cancer: Recent Insights. Cancers.

[31] Li Y., Seto E. (2016). HDACs and HDAC Inhibitors in Cancer Development and Therapy. Cold Spring Harb. Perspect. Med..

[32] Mrakovcic M., Kleinheinz J., Frohlich L.F. (2017). Histone Deacetylase Inhibitor-Induced Autophagy in Tumor Cells: Implications for p53. Int. J. Mol. Sci..

[33] Bolden J.E., Peart M.J., Johnstone R.W. (2006). Anticancer activities of histone deacetylase inhibitors. Nat. Rev. Drug Discov..

[34] Fotheringham S., Epping M.T., Stimson L., Khan O., Wood V., Pezzella F., Bernards R., La Thangue N.B. (2009). Genome-wide loss-of-function screen reveals an important role for the proteasome in HDAC inhibitor-induced apoptosis. Cancer Cell.

[35] Everix L., Seane E.N., Ebenhan T., Goethals I., Bolcaen J. (2023). Introducing HDAC-Targeting Radiopharmaceuticals for Glioblastoma Imaging and Therapy. Pharmaceuticals.

[36] Dokmanovic M., Clarke C., Marks P.A. (2007). Histone Deacetylase Inhibitors: Overview and Perspectives. Mol. Cancer Res..

[37] Smalley J.P., Cowley S.M., Hodgkinson J.T. (2020). Bifunctional HDAC Therapeutics: One Drug to Rule Them All?. Molecules.

[38] Rajak H., Singh A., Raghuwanshi K., Kumar R., Dewangan P.K., Veerasamy R., Sharma P.C., Dixit A., Mishra P. (2014). A structural insight into hydroxamic acid based histone deacetylase inhibitors for the presence of anticancer activity. Curr. Med. Chem..

[39] Zhang J., Zhong Q. (2014). Histone deacetylase inhibitors and cell death. Cell. Mol. Life Sci..

[40] Frew A.J., Johnstone R.W., Bolden J.E. (2009). Enhancing the apoptotic and therapeutic effects of HDAC inhibitors. Cancer Lett..

[41] Mrakovcic M., Kleinheinz J., Fröhlich L.F. (2019). p53 at the Crossroads between Different Types of HDAC Inhibitor-Mediated Cancer Cell Death. Int. J. Mol. Sci..

[42] Insinga A., Minucci S., Pelicci P.G. (2005). Mechanisms of selective anticancer action of histone deacetylase inhibitors. Cell Cycle.

[43] Peart M.J. (2003). Novel mechanisms of apoptosis induced by histone deacetylase inhibitors. Cancer Res..

[44] Gong P., Wang Y., Jing Y. (2019). Apoptosis Induction byHistone Deacetylase Inhibitors in Cancer Cells: Role of Ku70. Int. J. Mol. Sci..

[45] Bao L., Diao H., Dong N., Su X., Wang B., Mo Q., Yu H., Wang X., Chen C. (2016). Histone deacetylase inhibitor induces cell apoptosis and cycle arrest in lung cancer cells via mitochondrial injury and p53 up-acetylation. Cell Biol. Toxicol..

[46] Yang Z.J., Chee C.E., Huang S., Sinicrope F.A. (2011). The role of autophagy in cancer: Therapeutic implications. Mol. Cancer Ther..

[47] Shao Y., Gao Z., Marks P.A., Jiang X. (2004). Apoptotic and autophagic cell death induced by histone deacetylase inhibitors. Proc. Natl. Acad. Sci. USA.

[48] Rebecca V.W., Amaravadi R.K. (2016). Emerging strategies to effectively target autophagy in cancer. Oncogene.

[49] Kimmelman A.C. (2011). The dynamic nature of autophagy in cancer. Genes Dev..

[50] Pagotto A., Pilotto G., Mazzoldi E.L., Nicoletto M.O., Frezzini S., Pastò A., Amadori A. (2017). Autophagy inhibition reduces chemoresistance and tumorigenic potential of human ovarian cancer stem cells. Cell Death Dis..

[51] El-Khoury V., Pierson S., Szwarcbart E., Brons N.H., Roland O., Cherrier-De Wilde S., Plawny L., Van Dyck E., Berchem G. (2014). Disruption of autophagy by the histone deacetylase inhibitor MGCD0103 and its therapeutic implication in B-cell chronic lymphocytic leukemia. Leukemia.

[52] Gilardini Montani M.S., Granato M., Santoni C., Del Porto P., Merendino N., D’Orazi G., Faggioni A., Cirone M. (2017). Histone deacetylase inhibitors VPA and TSA induce apoptosis and autophagy in pancreatic cancer cells. Cell. Oncol..

[53] Richon V.M., Sandhoff T.W., Rifkind R.A., Marks P.A. (2000). Histone deacetylase inhibitor selectively induces p21WAF1 expression and gene-associated histone acetylation. Proc. Natl. Acad. Sci. USA.

[54] Dong Z., Yang Y., Liu S., Lu J., Huang B., Zhang Y. (2018). HDAC inhibitor PAC-320 induces G2/M cell cycle arrest and apoptosis in human prostate cancer. Oncotarget.

[55] Lee H.A., Chu K.B., Moon E.K., Kim S.S., Quan F.S. (2020). Sensitization to oxidative stress and G2/M cell cycle arrest by histone deacetylase inhibition in hepatocellular carcinoma cells. Free Radic. Biol. Med..

[56] Hrgovic I., Doll M., Kleemann J., Wang X.-F., Zoeller N., Pinter A., Kippenberger S., Kaufmann R., Meissner M. (2016). The histone deacetylase inhibitor trichostatin a decreases lymphangiogenesis by inducing apoptosis and cell cycle arrest via p21-dependent pathways. BMC Cancer.

[57] Schoepflin Z.R., Shapiro I.M., Risbud M.V. (2016). Class I and IIa HDACs Mediate HIF-1α Stability Through PHD2-Dependent Mechanism, While HDAC6, a Class IIb Member, Promotes HIF-1α Transcriptional Activity in Nucleus Pulposus Cells of the Intervertebral Disc. J. Bone Min. Res..

[58] Jeong J.W., Bae M.K., Ahn M.Y., Kim S.H., Sohn T.K., Bae M.H., Yoo M.A., Song E.J., Lee K.J., Kim K.W. (2002). Regulation and destabilization of HIF-1alpha by ARD1-mediated acetylation. Cell.

[59] Deroanne C.F., Bonjean K., Servotte S., Devy L., Colige A., Clausse N., Blacher S., Verdin E., Foidart J.M., Nusgens B.V. (2002). Histone deacetylases inhibitors as anti-angiogenic agents altering vascular endothelial growth factor signaling. Oncogene.

[60] Liu T., Kuljaca S., Tee A., Marshall G.M. (2006). Histone deacetylase inhibitors: Multifunctional anticancer agents. Cancer Treat. Rev..

[61] Iordache F., Buzila C., Constantinescu A., Andrei E., Maniu H. (2012). Histone deacetylase (HDAC) inhibitors down-regulate endothelial lineage commitment of umbilical cord blood derived endothelial progenitor cells. Int. J. Mol. Sci..

[62] Ellis L., Pili R. (2010). Histone Deacetylase Inhibitors: Advancing Therapeutic Strategies in Hematological and Solid Malignancies. Pharmaceuticals.

[63] Munshi A., Tanaka T., Hobbs M.L., Tucker S.L., Richon V.M., Meyn R.E. (2006). Vorinostat, a histone deacetylase inhibitor, enhances the response of human tumor cells to ionizing radiation through prolongation of γ-H2AX foci. Mol. Cancer Ther..

[64] Ediriweera M.K., Tennekoon K.H., Samarakoon S.R. (2019). Emerging role of histone deacetylase inhibitors as anti-breast-cancer agents. Drug Discov. Today.

[65] Luu T.H., Morgan R.J., Leong L., Lim D., McNamara M., Portnow J., Frankel P., Smith D.D., Doroshow J.H., Wong C. (2008). A phase II trial of vorinostat (suberoylanilide hydroxamic acid) in metastatic breast cancer: A California Cancer Consortium study. Clin. Cancer Res..

[66] Vitti E.T., Parsons J.L. (2019). The Radiobiological Effects of Proton Beam Therapy: Impact on DNA Damage and Repair. Cancers.

[67] Bian L., Meng Y., Zhang M., Li D. (2019). MRE11-RAD50-NBS1 complex alterations and DNA damage response: Implications for cancer treatment. Mol. Cancer.

[68] Hall E.J., Giaccia A.J. (2012). Radiobiology for the Radiologist.

[69] Fontana A.O., Augsburger M.A., Grosse N., Guckenberger M., Lomax A.J., Sartori A.A., Pruschy M.N. (2015). Differential DNA repair pathway choice in cancer cells after proton- and photon-irradiation. Radiother. Oncol..

[70] Kachhap S.K., Rosmus N., Collis S.J., Kortenhorst M.S., Wissing M.D., Hedayati M., Shabbeer S., Mendonca J., Deangelis J., Marchionni L. (2010). Downregulation of homologous recombination DNA repair genes by HDAC inhibition in prostate cancer is mediated through the E2F1 transcription factor. PLoS ONE.

[71] Bose P., Dai Y., Grant S. (2014). Histone deacetylase inhibitor (HDACI) mechanisms of action: Emerging insights. Pharmacol. Ther..

[72] Scotto L., Serrano X.J., Zullo K., Kinahan C., Deng C., Sawas A., Bates S., O’Connor O.A. (2020). ATM inhibition overcomes resistance to histone deacetylase inhibitor due to p21 induction and cell cycle arrest. Oncotarget.

[73] Brehm A., Miska E.A., McCance D.J., Reid J.L., Bannister A.J., Kouzarides T. (1998). Retinoblastoma protein recruits histone deacetylase to repress transcription. Nature.

[74] Ferreira R., Magnaghi-Jaulin L., Robin P., Harel-Bellan A., Trouche D. (1998). The three members of the pocket proteins family share the ability to repress E2F activity through recruitment of a histone deacetylase. Proc. Natl. Acad. Sci. USA.

[75] Barrett A., Shingare M.R., Rechtsteiner A., Wijeratne T.U., Rodriguez K.M., Rubin S.M., Müller G.A. (2023). HDAC activity is dispensable for repression of cell-cycle genes by DREAM and E2F:RB complexes. bioRxiv.

[76] Thurn K.T., Thomas S., Raha P., Qureshi I., Munster P.N. (2013). Histone deacetylase regulation of ATM-mediated DNA damage signaling. Mol. Cancer Ther..

[77] Miller K.M., Tjeertes J.V., Coates J., Legube G., Polo S.E., Britton S., Jackson S.P. (2010). Human HDAC1 and HDAC2 function in the DNA-damage response to promote DNA nonhomologous end-joining. Nat. Struct. Mol. Biol..

[78] Kao G.D., McKenna W.G., Guenther M.G., Muschel R.J., Lazar M.A., Yen T.J. (2003). Histone deacetylase 4 interacts with 53BP1 to mediate the DNA damage response. J. Cell Biol..

[79] Yamaguchi T., Cubizolles F., Zhang Y., Reichert N., Kohler H., Seiser C., Matthias P. (2010). Histone deacetylases 1 and 2 act in concert to promote the G1-to-S progression. Genes Dev..

[80] Hai R., He L., Shu G., Yin G. (2021). Characterization of Histone Deacetylase Mechanisms in Cancer Development. Front. Oncol..

[81] Valenzuela-Fernández A., Cabrero J.R., Serrador J.M., Sánchez-Madrid F. (2008). HDAC6: A key regulator of cytoskeleton, cell migration and cell-cell interactions. Trends Cell Biol..

[82] Bhaskara S., Knutson S.K., Jiang G., Chandrasekharan M.B., Wilson A.J., Zheng S., Yenamandra A., Locke K., Yuan J.L., Bonine-Summers A.R. (2010). Hdac3 is essential for the maintenance of chromatin structure and genome stability. Cancer Cell.

[83] Nishimoto K., Niida H., Uchida C., Ohhata T., Kitagawa K., Motegi A., Suda T., Kitagawa M. (2020). HDAC3 Is Required for XPC Recruitment and Nucleotide Excision Repair of DNA Damage Induced by UV Irradiation. Mol. Cancer Res..

[84] Zhang M., Xiang S., Joo H.Y., Wang L., Williams K.A., Liu W., Hu C., Tong D., Haakenson J., Wang C. (2014). HDAC6 Deacetylates and Ubiquitinates MSH2 to Maintain Proper Levels of MutSα. Mol. Cell.

[85] Wang R.-H. (2008). Impaired DNA Damage Response, Genome Instability, and Tumorigenesis in SIRT1 Mutant Mice. Cancer Cell.

[86] Polo S.E., Almouzni G. (2015). Chromatin dynamics after DNA damage: The legacy of the access-repair-restore model. DNA Repair.

[87] Fortuny A., Polo S.E. (2018). The response to DNA damage in heterochromatin domains. Chromosoma.

[88] Etier A., Dumetz F., Chéreau S., Ponts N. (2022). Post-Translational Modifications of Histones Are Versatile Regulators of Fungal Development and Secondary Metabolism. Toxins.

[89] Warters R.L., Lyons B.W. (1992). Variation in radiation-induced formation of DNA double-strand breaks as a function of chromatin structure. Radiat. Res..

[90] Cowell I.G., Sunter N.J., Singh P.B., Austin C.A., Durkacz B.W., Tilby M.J. (2007). gammaH2AX foci form preferentially in euchromatin after ionising-radiation. PLoS ONE.

[91] Kim J.A., Kruhlak M., Dotiwala F., Nussenzweig A., Haber J.E. (2007). Heterochromatin is refractory to gamma-H2AX modification in yeast and mammals. J. Cell Biol..

[92] Nygren J., Ljungman M., Ahnström M. (1995). Chromatin Structure and Radiation-induced DNA Strand Breaks in Human Cells: Soluble Scavengers and DNA-bound Proteins Offer a Better Protection Against Single- than Double-strand Breaks. Int. J. Radiat. Biol..

[93] Kruhlak M.J., Celeste A., Dellaire G., Fernandez-Capetillo O., Müller W.G., McNally J.G., Bazett-Jones D.P., Nussenzweig A. (2006). Changes in chromatin structure and mobility in living cells at sites of DNA double-strand breaks. J. Cell Biol..

[94] Lisby M., Mortensen U.H., Rothstein R. (2003). Colocalization of multiple DNA double-strand breaks at a single Rad52 repair centre. Nat. Cell Biol..

[95] Aten J.A., Stap J., Krawczyk P.M., van Oven C.H., Hoebe R.A., Essers J., Kanaar R. (2004). Dynamics of DNA double-strand breaks revealed by clustering of damaged chromosome domains. Science.

[96] Amaral N., Ryu T., Li X., Chiolo I. (2017). Nuclear Dynamics of Heterochromatin Repair. Trends Genet..

[97] Murr R., Loizou J.I., Yang Y.G., Cuenin C., Li H., Wang Z.Q., Herceg Z. (2006). Histone acetylation by Trrap-Tip60 modulates loading of repair proteins and repair of DNA double-strand breaks. Nat. Cell Biol..

[98] Xu Y., Sun Y., Jiang X., Ayrapetov M.K., Moskwa P., Yang S., Weinstock D.M., Price B.D. (2010). The p400 ATPase regulates nucleosome stability and chromatin ubiquitination during DNA repair. J. Cell Biol..

[99] Krawczyk P.M., Borovski T., Stap J., Cijsouw T., ten Cate R., Medema J.P., Kanaar R., Franken N.A., Aten J.A. (2012). Chromatin mobility is increased at sites of DNA double-strand breaks. J. Cell Sci..

[100] Kim J.H., Kim I.H., Shin J.H., Kim H.J., Kim I.A. (2013). Sequence-Dependent Radiosensitization of Histone Deacetylase Inhibitors Trichostatin A and SK-7041. Cancer Res. Treat..

[101] Van Nifterik K.A., Van den Berg J., Slotman B.J., Lafleur M.V., Sminia P., Stalpers L.J. (2012). Valproic acid sensitizes human glioma cells for temozolomide and gamma-radiation. J. Neurooncol..

[102] Moertl S., Payer S., Kell R., Winkler K., Anastasov N., Atkinson M.J. (2019). Comparison of Radiosensitization by HDAC Inhibitors CUDC-101 and SAHA in Pancreatic Cancer Cells. Int. J. Mol. Sci..

[103] Adimoolam S., Sirisawad M., Chen J., Thiemann P., Ford J.M., Buggy J.J. (2007). HDAC-inhibitor-PCI24781-decreases-RAD51-expression-and-inhibits-homologous-recombination. Proc. Natl. Acad. Sci. USA.

[104] Mueller S., Yang X., Sottero T.L., Gragg A., Prasad G., Polley M.Y., Weiss W.A., Matthay K.K., Davidoff A.M., DuBois S.G. (2011). Cooperation of the HDAC inhibitor vorinostat and radiation in metastatic neuroblastoma: Efficacy and underlying mechanisms. Cancer Lett..

[105] Flatmark K., Nome R.V., Folkvord S., Bratland A., Rasmussen H., Ellefsen M.S., Fodstad O., Ree A.H. (2006). Radiosensitization of colorectal carcinoma cell lines by histone deacetylase inhibition. Radiat. Oncol..

[106] Saelen M.G., Ree A.H., Kristian A., Fleten K.G., Furre T., Hektoen H.H., Flatmark K. (2012). Radiosensitization by the histone deacetylase inhibitor vorinostat under hypoxia and with capecitabine in experimental colorectal carcinoma. Radiat. Oncol..

[107] Shoji M., Ninomiya I., Makino I., Kinoshita J., Nakamura K., Oyama K., Nakagawara H., Fujita H., Tajima H., Takamura H. (2012). Valproic acid, a histone deacetylase inhibitor, enhances radiosensitivity in esophageal squamous cell carcinoma. Int. J. Oncol..

[108] Perona M., Thomasz L., Rossich L., Rodriguez C., Pisarev M.A., Rosemblit C., Cremaschi G.A., Dagrosa M.A., Juvenal G.J. (2018). Radiosensitivity enhancement of human thyroid carcinoma cells by the inhibitors of histone deacetylase sodium butyrate and valproic acid. Mol. Cell. Endocrinol..

[109] Oike T., Hirota Y., Dewi Maulany Darwis N., Shibata A., Ohno T. (2020). Comparison of Clonogenic Survival Data Obtained by Pre- and Post-Irradiation Methods. J. Pers. Med..

[110] Frankenberg-Schwager M., Frankenberg D., Harbich R. (1987). Potentially lethal damage repair is due to the difference of DNA double-strand break repair under immediate and delayed plating conditions. Radiat. Res..

[111] Reddy N.M., Kapiszewska M., Lange C.S. (1992). Detection of X-ray damage repair by the immediate versus delayed plating technique is dependent on cell shape and cell concentration. Scanning Microsc..

